# MCM8-9 complex promotes resection of double-strand break ends by MRE11-RAD50-NBS1 complex

**DOI:** 10.1038/ncomms8744

**Published:** 2015-07-28

**Authors:** Kyung Yong Lee, Jun-Sub Im, Etsuko Shibata, Jonghoon Park, Naofumi Handa, Stephen C. Kowalczykowski, Anindya Dutta

**Affiliations:** 1 Department of Biochemistry and Molecular Genetics, School of Medicine, University of Virginia, Jordan Hall, 1300 Jefferson Park Avenue, Charlottesville, Virginia 22908 USA; 2 Department of Microbiology and Molecular Genetics, University of California, Briggs Hall, One Shields Avenue, Davis, California 95616-8665 USA

## Abstract

MCM8-9 complex is required for homologous recombination (HR)-mediated repair of double-strand breaks (DSBs). Here we report that MCM8-9 is required for DNA resection by MRN (MRE11-RAD50-NBS1) at DSBs to generate ssDNA. MCM8-9 interacts with MRN and is required for the nuclease activity and stable association of MRN with DSBs. The ATPase motifs of MCM8-9 are required for recruitment of MRE11 to foci of DNA damage. Homozygous deletion of the *MCM9* found in various cancers sensitizes a cancer cell line to interstrand-crosslinking (ICL) agents. A cancer-derived point mutation or an SNP on *MCM8* associated with premature ovarian failure (POF) diminishes the functional activity of MCM8. Therefore, the MCM8-9 complex facilitates DNA resection by the MRN complex during HR repair, genetic or epigenetic inactivation of *MCM8* or *MCM9* are seen in human cancers, and genetic inactivation of *MCM8* may be the basis of a POF syndrome.

## Abstract (web summary)

The MCM8-9 complex is required for repair of DNA double-strand breaks (DSBs). Here, the authors show that MCM8-9 is required for the nuclease activity of the MRE11-RAD50-NBS1 complex (MRN) and stabilizes its association with DNA damage sites, promoting resection of DSB ends.

## Graphical abstract

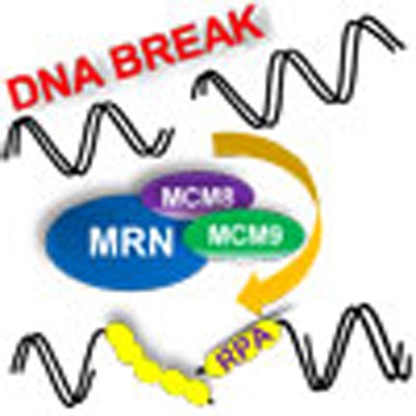

Double-strand break (DSB) repair is essential for the maintenance of DNA integrity^1^. Deregulation of this process leads to significant genetic instability, which can result in the development of tumours^2^. DSB repair systems are largely classified into homologous recombination (HR) and non-homologous end joining (NHEJ). In the first step of HR repair, MRN (MRE11-RAD50-NBS1) complex and C-terminal binding protein (CtBP)-interacting protein (CtIP) recognize DNA breaks, resect single-stranded DNA (ssDNA) together with BLM/Dna2 and Exo1, and thus generate a long stretch of 3′-overhanging ssDNA^3^,^4^,^5^,^6^. Following DNA resection, RPA proteins are recruited to the ssDNA to stabilize the structure, and mediator proteins, including Rad51, Rad52 and BRCA2, promote the formation of Rad51 filaments^7^. Recent papers show that the endonuclease activity of MRE11 in the MRN complex distinguishes HR from NHEJ^8^,^9^.

Interstrand-crosslinking (ICL)-inducing chemotherapy agents such as cisplatin and mitomycin C produce lesions that are repaired by HR^10^,^11^. Hence, inactivation of HR makes cells very sensitive to ICL adducts, and cancers defective in HR (for example, those with *BRCA1* or *BRCA2* mutations) are good targets for treatment with cisplatin or mitomycin C. HR, as an important part of meiosis, is also very important for the generation of germ cells.

Even though MCM8-9 proteins have initially been identified as components of pre-replication complexes, recent findings counter the original suggestion that MCM8-9 is essential for DNA replication. The *Xenopus* MCM8 clearly shows DNA helicase activity *in vitro*^12^, and mutants of the *Drosophila* MCM8 homologue, REC, have meiotic crossover defects^13^. However, mice with homozygous deletions of MCM9 are viable and fertile, albeit with some deficits in the germ cell lineage, a lineage notable for meiosis^14^. Mouse and chicken cell lines with deletions of *MCM8* or *MCM9* are viable, although more sensitive to cisplatin 15,16,17. A single-nucleotide polymorphism (SNP; rs16991615) that leads to an amino-acid change from glutamic acid (Glu) to lysine (Lys) in the gene *MCM8* was found in genome-wide association analysis to be significantly correlated with age at natural menopause^18^. As a whole, the evidence suggests that MCM8-9 is more important for HR than for DNA replication, but the exact role of MCM8-9 in HR is unclear.

Here we show that MCM8-9 is essential for DNA resection by the MRN complex at DSBs, and is required for proper localization of the MRN complex to the DSBs. In addition, a cancer cell line having homozygous deletion of the *MCM9* locus exhibits inefficient HR and high sensitivity to ICL reagents. Inactivation of the MCM8-9 complex is seen in cancers and in a premature ovarian failure (POF). Genetic or epigenetic inactivation of MCM8-9 is a new mechanism by which cancers can blunt the HR repair pathway, and as in cancers with mutations in *BRCA1* or *BRCA2*, cancers that inactivate MCM8-9 are susceptible to chemotherapy agents that target cells with blunted HR.

## Results

### MCM8-9 is required for ssDNA generation in HR repair

**Figure 1:**
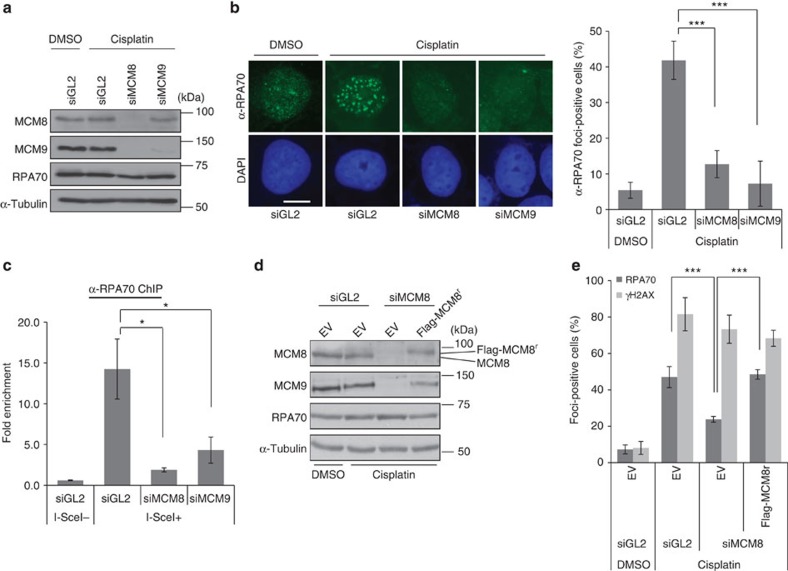
MCM8-9 is required for RPA binding to DSB sites. (**a**) Immunoblots of lysates from U2OS cells transfected with indicated siRNAs, 4 h after addition of cisplatin. (**b**) Decrease of RPA70 foci on depletion of MCM8 or MCM9. RPA70 immunofluorescence images shown at left and cells having over 20 foci of RPA70 were counted as positive cells on the right. Scale bar, 10 μm. ***P<0.005; Student's *t*-test. (**c**) ChIP assay in HeLa DR13-9 cells. Fold signal of RPA70 at I-SceI cut site relative to control site 2 kb upstream of cut site in cell treated with indicated siRNAs. *P<0.05; Student's *t*-test. (**d**) Immunoblot of U2OS cells stably transfected with empty vector (EV) or plasmid expressing siMCM8 resistant, Flag-MCM8r, 48 h after transfection of siRNAs. (**e**) Restoration of RPA foci formation by siMCM8-resistant Flag-MCM8. Quantification of RPA70 or γH2AX foci-positive cells as in Fig. 1b. ***P<0.005; Student's *t*-test. All error bars represent s.d. of the mean from triplicates. DAPI, 4′,6′-diamidino-2-phenylindole.

Deficiency of MCM8 or MCM9 impairs the recruitment of Rad51 on chromatin after DNA damage^15^,^17^. DNA resection by the MRN complex, CtIP, EXO1 and DNA2, produces the ssDNA that has to be coated by RPA before subsequent Rad51 assembly^19^,^20^. We thus measured the accumulation of RPA at DNA damage sites in MCM8- or MCM9-depleted cells. As reported previously, depletion of MCM8 decreases MCM9, but depletion of MCM9 does not deplete MCM8 (Fig. 1a). Even though levels of RPA70 were unchanged, there was a decrease in cisplatin-induced RPA foci formation on MCM8 or MCM9 depletion (Fig. 1a,b). HeLa DR13-9 cells contain a single I-SceI cut site integrated into their genome that is repaired by HR after cleavage by the I-SceI^21^. Although the expression level of I-SceI was not diminished in MCM8- or MCM9-depleted cells (Supplementary Fig. 1), RPA binding to the I-SceI cut site was decreased in cells depleted of MCM8 or MCM9 compared with control short interfering RNA (siRNA; siGL2)-transfected cells (Fig. 1c). To eliminate the possibility of off-target effects of siRNAs, we rescued cisplatin-induced RPA foci formation in siMCM8-transfected cells by stably expressing siRNA-resistant Flag-tagged MCM8 (Flag-MCM8r; Fig. 1d,e). H2AX phosphorylation on S139, and by inference the number of DNA breaks, was not affected by MCM8-9 depletion, suggesting that MCM8-9 specifically affected a step after DNA break formation.

**Figure 2:**
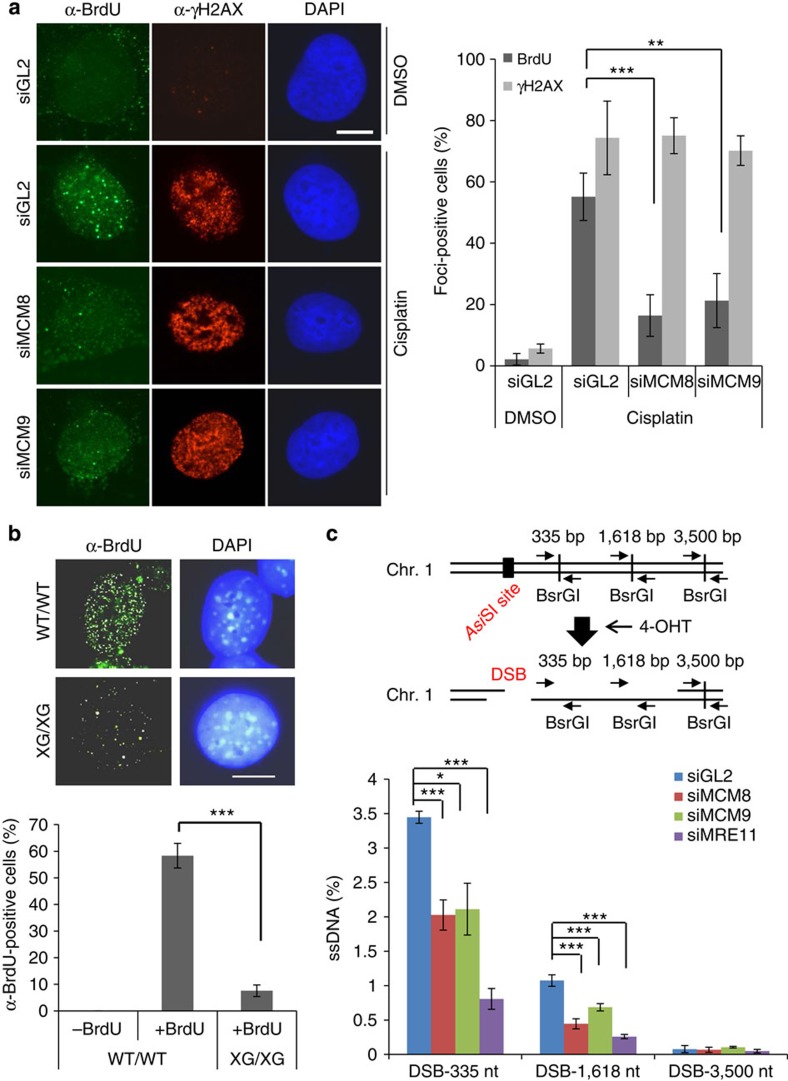
ssDNA generation at DSB depends on MCM8 or MCM9. (**a**) ssDNA foci measured by BrdU staining without DNA denaturation and γH2AX immunofluorescence foci. Representative images on left and quantification of foci-positive cells on the right. Scale bar, 10 μm. ***P<0.005, **P<0.01; Student's *t*-test. (**b**) ssDNA foci (BrdU foci) in MEFs with WT *MCM9* (WT/WT) or with homozygous mutation for *MCM9* (XG/XG) after exposure to cisplatin. Bottom: mean±s.d. of triplicates. Scale bar, 10 μm. ***P<0.005; Student's *t*-test. (**c**) Quantitative measurement of DNA resection 4 h after addition of 4-OHT to ER-AsiSI U2OS cells. Percentage of ssDNA at indicated sites was measured by qPCR using the primer pairs indicated on the cartoon after digestion with BsrGI. Bottom: % of ssDNA at different sites. ***P<0.005, *P<0.05; Student's *t*-test. All error bars represent s.d. of the mean from triplicates.

To directly examine whether MCM8-9 is required for ssDNA generation, the generation of ssDNA at DSB sites was directly visualized by immunostaining with anti-BrdU (5-bromodeoxyuridine) antibody without denaturation of the double-stranded DNA after labelling of genomic DNA with BrdU in a previous cell cycle. Cisplatin increased the number of cells with BrdU signal, but this was decreased by depletion of MCM8 or MCM9 (Fig. 2a). Similarly, cisplatin treatment of mouse embryonic fibroblast (MEF) cells from *Mcm9*-null (XG/XG) mice produced fewer ssDNA-positive cells compared with wild-type (WT) MEFs (Fig. 2b). Cell cycle profile of the MEF cells from XG/XG mice was not changed by the 4-h cisplatin treatment, suggesting that the decrease in ssDNA formation could not be explained by a change in S phase progression in these cells (Supplementary Fig. 2). Next, we utilized a new assay for quantitatively measuring ssDNA at regions adjoining a specific DSB site induced by the restriction enzyme *Asi*SI in the estrogen receptor (ER)-*Asi*SI U2OS cells^22^. The formation of ssDNA makes BsrG1 sites in the adjoining DNA resistant to digestion by that enzyme, thus allowing us to measure whether the end resection complex has digested past each of the three BsrGI site. DSB induced by 4-hydroxytamoxifen (4-OHT) treatment resulted in more ssDNA at the BsrG1 site closer to *Asi*SI cut site, but at both this site and the next, depletion of MCM8 or MCM9 decreased the generation of ssDNA (Fig. 2c). Depletion of MRE11 also affected ssDNA formation at these sites, validating the assay. Therefore MCM8-9 is required for ssDNA formation at DSBs before HR.

### MCM8-9 is required for MRN localization to HR repair sites

**Figure 3:**
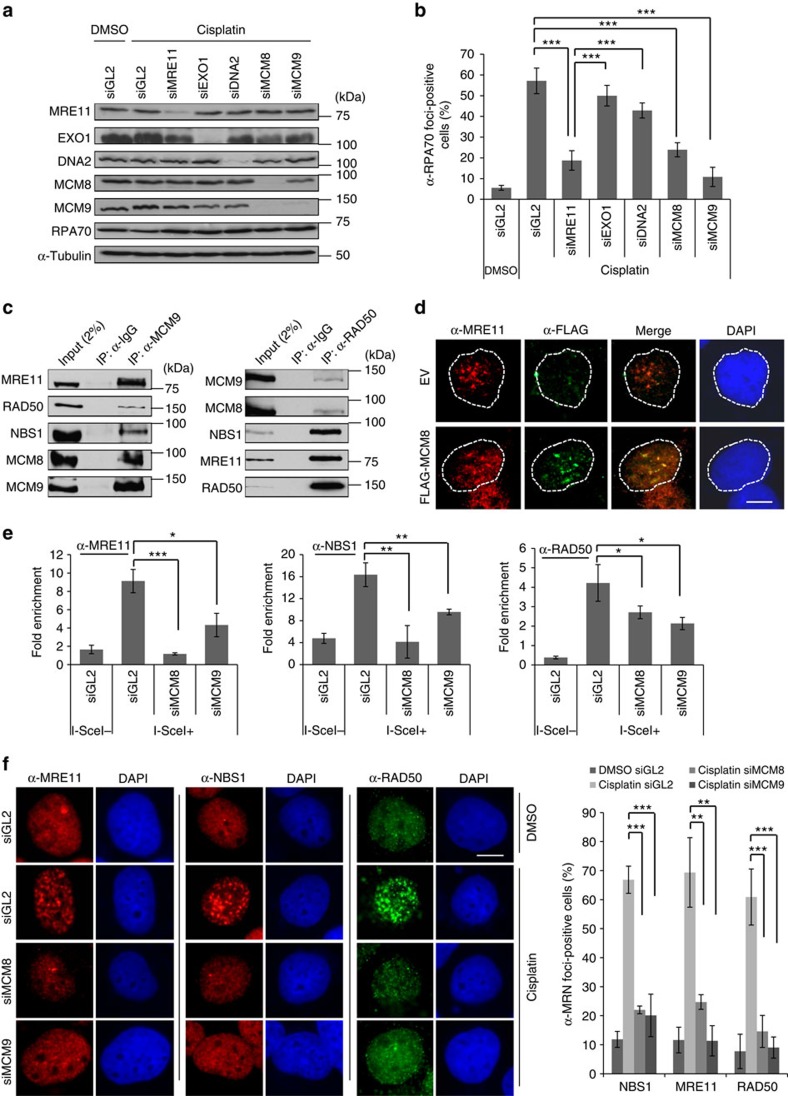
MCM8-9 are required for the localization of MRN complex on HR repair sites. (**a**,**b**) MCM8-9 and MRE11 required for forming RPA foci in cisplatin-treated cells. The western blot (**a**) and quantification of RPA70 foci-positive cells (**b**) after knockdown of indicated proteins in U2OS cells. ***P<0.005; Student's *t*-test. (**c**) MRN complex coimmunoprecipitates with MCM8-9. Endogenous MCM9 (left) or Rad50 (right) was immunoprecipitated (IP) from HEK293T cells using indicated antibodies in the presence of EtBr and immunoblotted for indicated proteins. (**d**) Co-localization of Flag-MCM8 and MRE11 in nuclear foci after exposure to cisplatin. Cells were pre-extracted for immunostaining. Scale bar, 10 μm. (**e**) Defect of MRN recruitment to I-SceI cut site in MCM8- or MCM9-depleted cells. ChIP assays were performed using indicated antibodies in HeLa DR13-9 cells 18 h after transfecting plasmid expressing I-SceI. Fold signal at cut site relative to site 2 kb upstream as described in Fig. 1c. ***P<0.005, **P<0.01, *P<0.05; Student's *t*-test. (**f**) Decrease of MRN foci-positive cells in MCM8- or MCM9-depleted cells. Representative images (left) and % of MRN foci-positive cells (right). Cells having over 20 foci >0.5 μm diameter were counted as positive. Scale bar, 10 μm. ***P<0.005, **P<0.01; Student's *t*-test. All error bars represent s.d. of the mean from triplicates. DAPI, 4′,6′-diamidino-2-phenylindole; EV, empty vector; IgG, immunoglobulin G.

To screen for the nuclease(s) requiring MCM8-9 activity for ssDNA formation, we first tested which nucleases were required for RPA focus formation after cisplatin treatment. The knockdown of MRE11, but not EXO1 and/or DNA2, suppressed cisplatin-induced RPA foci formation to the same extent as seen after MCM8-9 depletion (Fig. 3a,b and Supplementary Fig. 3a,b). This result suggested that MCM8-9 might work during the initial DNA resection alongside the MRN complex. Therefore, we examined whether MRN and MCM8-9 physically associate with each other. Indeed, MCM9 immunoprecipitates contained not only MCM8 but also MRE11, NBS1 and RAD50 (Fig. 3c, left), and conversely RAD50 immunoprecipitates contained not only its cofactors, NBS1 and MRE11, but also MCM8-9 (Fig. 3c, right). The co-immunoprecipitation was performed in the presence of the DNA intercalating chemical, ethidium bromide (EtBr), suggesting that the MCM8-9 and MRN did not interact via a bridging DNA molecule. Furthermore, MRE11 foci generated by cisplatin treatment co-localized with Flag-MCM8 foci (Fig. 3d). Chromatin immunoprecipitation (ChIP) assays on HeLa DR13-9 cells showed that all of the MRN components normally accumulated on the I-SceI cut site following the expression of I-SceI, but this association was impaired on depletion of MCM8 or MCM9 (Fig. 3e). In a parallel experiment, siMCM8 or siMCM9 decreased the number of cisplatin-induced foci formed by all MRN complex members (Fig. 3f). On the other hand, the recruitment of CtIP to cisplatin induced foci and to I-SceI cut sites was not affected by depletion of MCM8-9 (Supplementary Fig. 4a,b). Therefore, in multiple experimental systems, MCM8-9 is required for the proper localization of the MRN complex to DSB sites for DNA resection.

### ATPase activity of MCM9 is essential for the function of MRN

**Figure 4:**
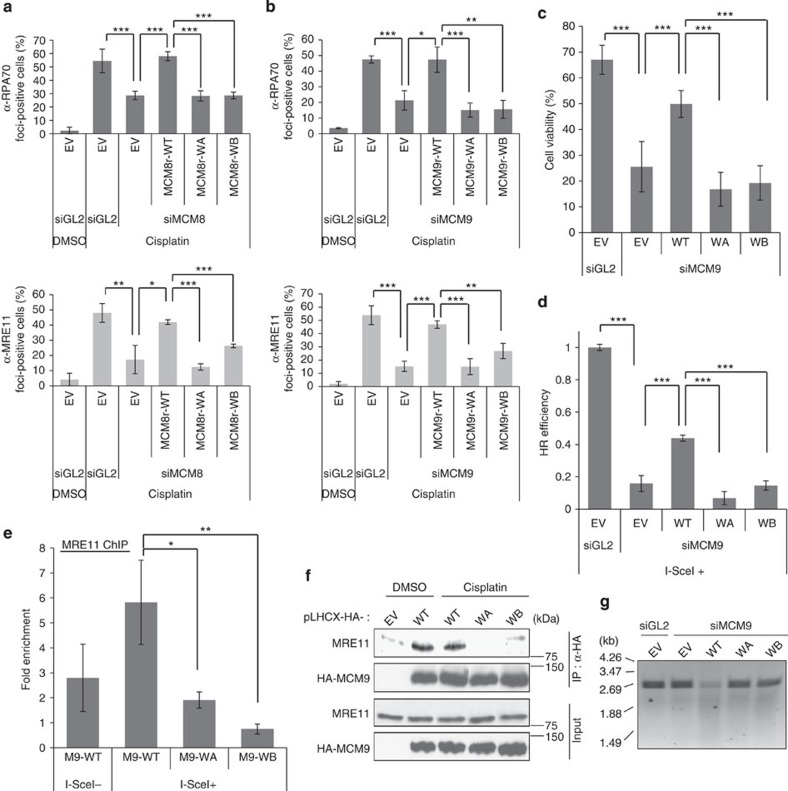
ATPase motif of MCM9 is essential for HR repair and the interaction with MRE11 protein. (**a**,**b**) Mutation on ATPase motif of MCM8-9 decreases RPA (top) or MRE11 (bottom) foci formation. U2OS cells supported by Walker A- (WA) or Walker B (WB) mutants of MCM8 (**a**) or MCM9 (**b**) were treated with cisplatin after knockdown of the endogenous protein, and foci-positive cells were counted as described previously. ***P<0.005, **P<0.01, *P<0.05; Student's *t*-test. (**c**) WA- or WB mutant of MCM9 cannot restore resistance to cisplatin after knockdown of endogenous MCM9. Cell viability was measured by colony count at day 5 after cisplatin treatment . ***P<0.005; Student's *t*-test. (**d**) WA- or WB mutant MCM9 cannot rescue HR. HR assays were performed in HeLa DR13-9 cells having stable expression of siRNA-resistant MCM9. HR efficiency was measured by normalizing the percentage of GFP-positive cells of each sample to that of the siGL2-treated cells. ***P<0.005; Student's *t*-test. (**e**) WA- or WB mutant MCM9 cannot recruit MRE11 to I-SceI cut site. ChIP was done using HeLa DR13-9 cells having stable expression of siRNA-resistant MCM9 after knockdown of endogenous MCM9. Signal at cut site expressed relative to -2 kb site. **P<0.01, *P<0.05; Student's *t*-test. (**f**) WA- or WB mutant MCM9 does not co-immunoprecipitate MRE11 from HEK293T cells transfected by the indicated plasmids expressing MCM9. (**g**) Decrease in nuclease associated with WA- or WB mutant MCM9. DNA products visualized after *in vitro* nuclease assay for 90 min with epitope-tagged MCM9 immunoprecipitated (IP) from cells transfected with indicated plasmids and siRNAs as described in Methods section. Cells were treated with 40 μM cisplatin for 4 h before harvest. All error bars represent s.d. of the mean from triplicates. EV, empty vector; WT, wild type.

MCM8 and MCM9 are related in sequence to all of the components of the MCM2-7 complex, which unwinds duplex DNA during DNA replication^23^. Human MCM8 and MCM9 contain MCM domains marked by the Walker A (WA) and Walker B (WB) motifs important for ATP binding and hydrolysis. Therefore, we tested whether the ATPase activities of MCM8 or MCM9 were essential for DNA resection in HR repair. U2OS cell lines stably expressing WA or WB motif mutants of siRNA-resistant MCM8 or MCM9 were used. On depletion of endogenous MCM8 or MCM9 by siRNA, WT MCM8 and MCM9 (MCM8r-WT and MCM9r-WT) restored the RPA- and MRE11 foci on cisplatin treatment (Fig. 4a,b). The WA- or WB motif mutants of MCM8 or MCM9 were unable to restore these foci.

Because knockdown of MCM8 decreased both MCM8 and MCM9, while knockdown of MCM9 decreases that protein alone, we focused on the MCM9 mutants from here on. The recruitment of the DNA damage response kinase ATR/ATRIP to damaged DNA is mediated through its interaction with RPA, which is necessary for proper checkpoint activation^24^,^25^,^26^. Consistent with a role of MCM9 in ssDNA formation and RPA recruitment, phospho-Chk1 was decreased in siMCM9-transfected cells, and phospho-Chk1 was restored following expression of MCM9r-WT, but not WA- or WB mutants of MCM9 (Supplementary Fig. 5). The WA- or WB mutants of MCM9 may even have a dominant-negative effect on checkpoint activation, perhaps because they interact with endogenous MCM8 and suppress any residual activity from MCM8 alone. The sensitivity of the cells to cisplatin was increased by siMCM9, restored by WT MCM9, but not by WA- or WB mutants of MCM9, suggesting that the ATPase activity of MCM9 is critical for cell survival following treatment with ICL-inducing agents (Fig. 4c and Supplementary Fig. 6). WT MCM9 partly restored HR efficiency to HeLa DR13-9 cells depleted of endogenous MCM9, whereas WA- or WB mutants of MCM9 did not (Fig. 4d).

Next, we examined whether the ATPase activity of MCM9 is necessary for MRE11 recruitment to DSBs. In ChIP experiments in HeLa DR13-9 cells, MRE11 recruitment to I-SceI cut sites was not rescued by MCM9 with mutations in WA or B motifs (Fig. 4e). The co-immunoprecipitation of MRE11 with MCM9 was also inhibited by the same mutations (Fig. 4f). Furthermore, *in vitro* nuclease assay on linearized pUC19 plasmid using purified HA-MCM9 from HeLa DR13-9 cells suggested that only MCM9 WT was associated with a strong nuclease activity (Fig. 4g).

**Figure 5:**
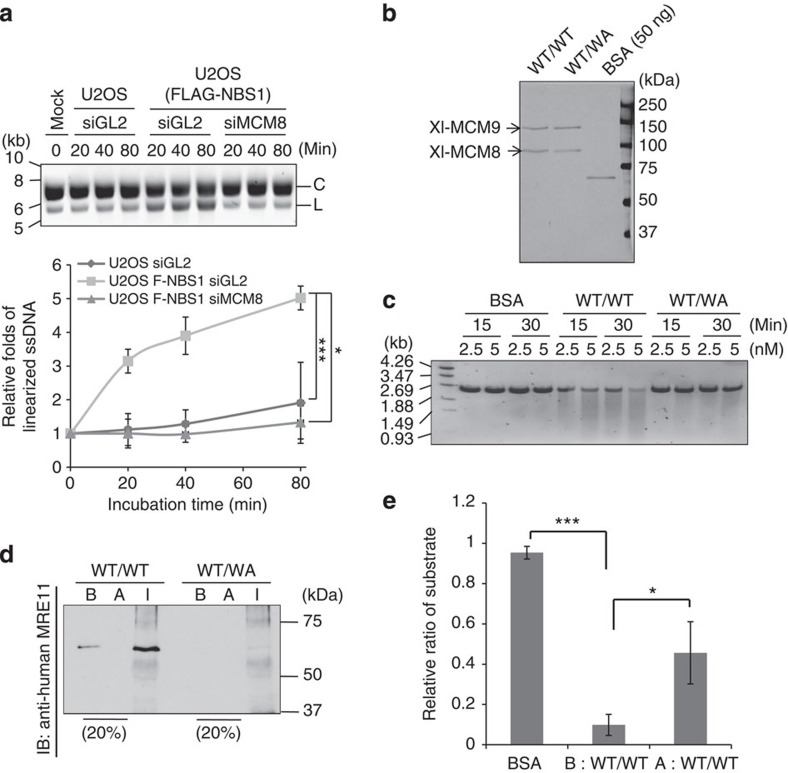
ATPase activity of MCM9 is essential for the function of MRN nuclease. (**a**) Decrease of MRN endonuclease activity in MCM8-9-depleted cells. *In vitro* endonuclease assay using MRN purified by anti-FLAG from U2OS cells stably expressing FLAG-NBS1. ØX174 ssDNA substrate was incubated for indicated times. Top: EtBr stain of reaction products shows the substrate C, circular ØX174 and the product L, linearized ØX174. Bottom: quantification of linearized ssDNA with ImageJ software, normalized to the level in the 0-min lane. ***P<0.005, *P<0.05; Student's *t*-test. (**b**) Silver stain of purified recombinant *Xenopus* MCM8-9 complex. WT, wild type-MCM8 and -MCM9; WA, WA mutant MCM9. (**c**) WT/WT MCM8-9 has more nuclease activity than WT/WA MCM8-9. The *in vitro* nuclease assay was performed using indicated amounts of the recombinant MCM8-9 or bovine serum albumin (BSA). (**d**) Immunodepletion of MRE11, as detected by immunoblot, from purified recombinant MCM8-9. Immunodepletion was done by incubating anti-human MRE11 antibody with indicated recombinant proteins. B, before immunodepletion; A, after immunodepletion; I, the total immunoprecipitate. (**e**) Reduced nuclease activity of WT/WT MCM8-9 after immunodepletion of MRE11 (A), compared with that before immunodepletion (B). Amount of full-length linear DNA remaining was quantified after 60 min of an *in vitro* nuclease assay with 5 nM of BSA or purified MCM8-9 before or after immunodepletion of MRE11. The *y* axis shows the ratio of the residual substrate relative to that at the 0-min point. ***P<0.005, *P<0.05; Student's *t*-test. All error bars represent s.d. of the mean from triplicates.

MRE11 endonuclease initiates resection at DSBs before HR^8^. We purified the MRN complex from U2OS cells stably expressing FLAG-NBS1 and tested its endonuclease activity on circular ØX174 ssDNA (Fig. 5a and Supplementary Fig. 7a). MCM8 knockdown decreased the endonuclease activity of the immunoprecipitated MRN proteins, suggesting that human MCM8-9 is required for optimal nuclease activity of the MRN complex. Note that the nuclease activity of purified MRN complex was inhibited by MRE11 inhibitor, mirin (Supplementary Fig 7b). Thus, the nuclease activity in the MRN immunoprecipitate was mainly due to MRE11, although we cannot rule out the presence of other contaminating endonucleases.

Next, we purified recombinant *Xenopus* MCM8-MCM9 WT (WT/WT) from baculovirus-infected insect cells to investigate whether MCM8-9 can stimulate MRE11 endonuclease *in vitro* (Fig. 5b). MCM8 WT MCM9 WA mutant (WT/WA) complexes were to be used as inactive controls. We were thwarted from doing the experiment by a nuclease that was associated with the purified WT/WT MCM8-9 but not with the inactive WT/WA MCM8-9 (Fig. 5c). Since the WA mutation of human MCM9 decreased its association with human MRE11 (Fig. 4f), we wondered whether insect MRE11 was co-purifying with the Xl-WT/WT MCM8-9 complex. Remarkably, a 65-kDa protein, corresponding in size to *Drosophila* MRE11 and recognized by anti-MRE11 antibody, was associated with WT/WT but not WT/WA MCM8-9 complex (Fig. 5d, the B lanes). Anti-MRE11 antibody immunodepleted the 65-kDa protein (Fig. 5d, the A and I lanes) and decreased the nuclease activity associated with MCM8-9 (Fig. 5e). Several controls showed that the WA mutation of MCM9 did not decrease MCM8-9 complex formation (Supplementary Fig. 8), but decreased the ATPase activity of MCM8-9 (Supplementary Fig. 9), and that the MRE11 antibody decreased the nuclease associated with the recombinant Xl-MCM8-9 without decreasing the amount of MCM8-9 proteins (Supplementary Fig. 10).

These results suggest that ATPase activity of MCM8-9 complex is required for ssDNA generation at DSBs as well as optimal nuclease activity of the MRN. Also, ATP binding and hydrolysis by the MCM9 in the MCM8-9 complex is essential for maximal association with Mre11 and for the recruitment of MRN to DNA damage sites.

### Functional inactivation of MCM9 in cancers

**Figure 6:**
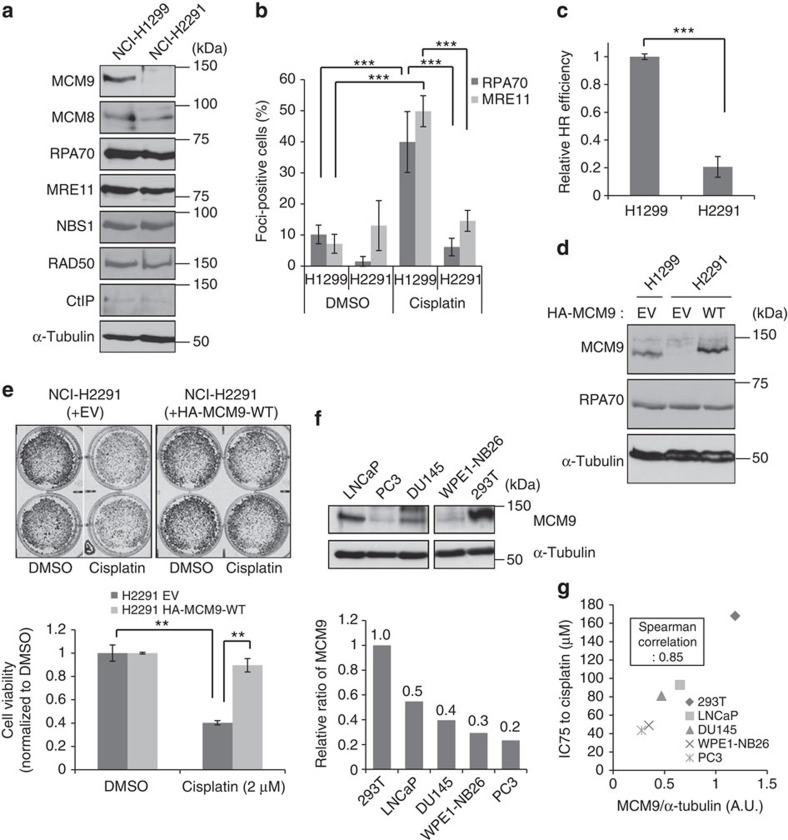
Functional inactivation of MCM9 in cancers. (**a**) Absence of MCM9 protein in NCI-H2291. Immunoblot of indicated proteins in lysates of NCI-H2291 and NCI-H1299 (control cell line). (**b**) Decrease of cisplatin-induced RPA70- or Mre11 foci-positive cells in NCI-H2291. ***P<0.005; Student's *t*-test. (**c**) Decrease of HR efficiency in NCI-H2291. HR assay was performed by transient transfection of DSB recombination reporter and I-SceI expression plasmids as described in Methods section. HR efficiency was calculated by normalizing the percentage of GFP-positive cells to transfection efficiency in each cell line. ***P<0.005; Student's *t*-test. (**d**) Transient expression of ectopic HA-MCM9 (WT) in NCI-H2291. (**e**) Restoration of relative resistance of NCI-H2291 to cisplatin by overexpression of MCM9. Cell viability was measured by clonogenic assay as described in Methods section. Top: representative wells. Bottom: quantification of viable cells. **P<0.01; Student's *t*-test. (**f**) Protein expression of MCM9 in prostate cancer cells. Top: amount of MCM9 protein in each cancer cell line measured by immunoblotting. All lanes were in the same blot and exposed similarly. Bottom: MCM9 signal quantified with ImageJ software, normalized to α-tubulin and expressed relative to 293T. (**g**) Correlation of MCM9 levels to IC75 to cisplatin in indicated cancer cell lines (also see viability curves measured by MTT assay in Supplementary Fig. 12). All error bars represent s.d. of the mean from triplicates. EV, empty vector.

Human cancers frequently show homo- and heterozygous deletions or translocations on 6q22.31, the genomic region containing the *MCM9* gene^27^,^28^,^29^. Copy-number variation studies in cancer genomes collated at CBioPortal^30^, and show that 6–7% of prostate cancers and salivary adenoid cystic carcinomas, and a smaller fraction of other cancers have homozygous deletion of *MCM9* (examples listed in Table 1). Several cancer cell lines, including a non-small cell lung cancer cell line, NCI-H2291, also have a homozygous deletion of the *MCM9* locus. To test whether the cancer-specific loss of *MCM9* affected HR repair, we compared the ability of NCI-H2291 and NCI-H1299, a control non-small cell lung cancer cell line, to cope with cisplatin-induced DNA damage. Although protein levels of MCM8 and other HR repair factors were similar, MCM9 protein was not detected in NCI-H2291 (Fig. 6a). The NCI-H2291 cells showed fewer cells positive for RPA and MRE11 foci following cisplatin treatment (Fig. 6b). Furthermore, NCI-2291 showed significant defect on HR efficiency compared with NCI-1299 in an HR assay based on transient transfection of the HR reporter and I-SceI expressing plasmids (Fig. 6c). Complementation with hemagglutinin (HA)-tagged WT MCM9 (HA-MCM9-WT; Fig. 6d) rescued RPA foci formation (nuclei with HA-MCM9 in Supplementary Fig. 11). Clonogenic assays showed that the deletion of *MCM9* in NCI-H2291 cells also made them more sensitive to cisplatin than those complemented with HA-MCM9-WT (Fig. 6e). Thus, deletion of *MCM9* produces a functional defect of HR repair in this cancer cell line. In addition, we found that several prostate cancer cell lines do not have deletions in *MCM9*, but express lower levels of the MCM9 protein compared with HEK293T cells (Fig. 6f). The cisplatin resistance of these prostate cancer cell lines was remarkably correlated to the amount of MCM9 protein expressed (Fig. 6g and Supplementary Fig. 12). Therefore, epigenetic suppression of MCM9 could also predispose cancer cells to cisplatin sensitivity.

### Functional inactivation of MCM8 by mutation in cancer or POF

**Figure 7:**
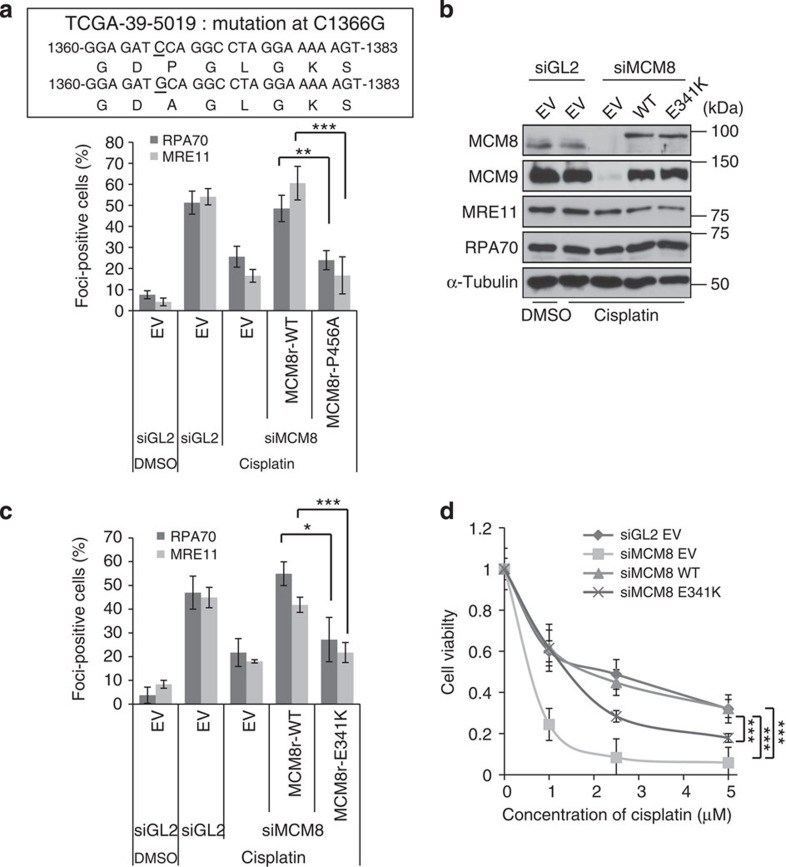
MCM8 point mutation in cancer and in POF. (**a**) Decrease of cisplatin-induced RPA70- or Mre11 focus formation in cells supported by P456A-MCM8 after knockdown of endogenous WT MCM8. The genetic mutation seen in certain squamous cell carcinomas is depicted in the upper panel and quantification of foci-positive cells in the lower panel as previously described. ***P<0.005, **P<0.01; Student's *t*-test. (**b**–**d**) Functional defect of the E341K mutant of *MCM8* associated with POF. (**b**) Immunoblot of U2OS cells stably expressing E341K mutant of MCM8, 48 h after transfection of siRNAs. (**c**) Decrease of cisplatin-induced RPA70- and MRE11 focus formation in E341K mutant of MCM8. RPA70 or MRE11 foci-positive cells were quantified after treatment with 40 μM cisplatin for 4 h as previously described. ***P<0.005, *P<0.05; Student's *t*-test. (**d**) E341K mutation on MCM8 renders cells more sensitive to cisplatin. Cell viability was measured by clonogenic assay. ***P<0.005; Student's *t*-test. All error bars represent s.d. of the mean from triplicates.

Finally, we turned to a lung squamous cell carcinoma in CBioPortal, with a point mutation that changed proline 456 in the WA motif of MCM8 to alanine (Fig. 7a, the upper panel). Functional studies in a cell line stably expressing siRNA-resistant MCM8 P456A showed that this mutation inactivated MCM8 because there was a decrease of cisplatin-induced RPA70- and MRE11 foci on knockdown of endogenous MCM8 (Fig. 7a, lower panel). Thus, genetic and epigenetic inactivation of MCM8-9 is seen in diverse human cancers, and such inactivation is associated with sensitivity to therapy by ICL agents.

Genome-wide association studies (GWAS) identified an SNP on *MCM8* leading to an amino-acid change (E341K) that is associated with an early age of natural menopause^18^ (a POF syndrome). MCM8 E341K was not able to rescue the RPA70- and MRE11 foci formation seen after depletion of endogenous MCM8 (Fig. 7b,c) nor could it fully restore the sensitivity of the MCM8-depleted cells to cisplatin (Fig. 7d). Consistent with a previous result^31^, the naturally occurring SNP of *MCM8* associated with POF impairs the function of MCM8 in HR repair. Since HR repair is an essential function of meiosis, mutational inactivation of MCM8 may explain the genetic basis of this POF syndrome.

## Discussion

DNA end resection is more pronounced in HR than in classical NHEJ^1^. In this study, we show that MCM8-9 is required for ssDNA generation at DSBs using the following three independent approaches: (a) detection of RPA70, (b) exposure of BrdU-labelled DNA and (c) quantitative PCR (qPCR)-based assay to measure ssDNA adjoining a DSB (Figs 1 and 2). Several DNA nucleases have been implicated in the resection: MRN complex and CtIP for the initial resection/EXO1 and DNA2 for the longer stretches of ssDNA^3^,^32^,^33^,^34^,^35^,^36^. It has also been suggested that MRE11 endonuclease initiates resection followed by bidirectional exonuclease activity of MRE11 and EXO1 to generate ssDNA for HR^8^. In our hands, EXO1 and DNA2 appear dispensable for RPA focus formation after cisplatin treatment, in contrast to the requirement of MRE11 (Fig. 3b and Supplementary Fig. 3b), perhaps because the secondary and extensive DNA resection attributed to EXO1 and DNA2 is slower than that caused by MRE11, and is not necessary for forming RPA foci. This does not mean that EXO1 and DNA2 are totally dispensable for resection *in vivo*. However, the parallel effects of MCM8-9 and MRE11 depletion on RPA focus formation is consistent with our suggestion that MCM8-9 is particularly involved in the initial resection by MRE11.

MCM8-9 recruits or promotes the stable association of MRN to the DSB (Fig. 3), without much effect on CtIP recruitment. This suggests that CtIP can be recruited to DSBs independent of MRN, which is in line with a previous report^37^. Also, since the MRN complex directly binds to DNA ends *in vitro*^38^, these results suggest either that MCM8-9 is required in cells on top of the direct DNA binding to recruit MRN, or that MCM8-9 prevents the detachment of active MRN after it has been recruited. MCM8-9 has highly conserved ATPase motifs and shows a helicase activity *in vitro*^12^,^39^. We show that mutation of its ATPase motif causes loss of interaction with MRN complex, increase of cellular sensitivity to cisplatin as well as decrease of HR efficiency (Figs 4 and 5). If anything, the mutant forms of MCM9 appear to have a dominant-negative effect on checkpoint activation (an indirect measure of ssDNA formation), HR and cell survival after cisplatin treatment (Supplementary Fig. 5 and Fig. 4c,d). The WA- or WB mutants of MCM9 most likely associate with endogenous MCM8 or other endogenous proteins to inactivate them further than seen after simple depletion of MCM9.

MCM8-9 ATPase could be a new therapeutic target whose inhibition would impair HR and augment the efficacy of ICL-inducing agents during chemotherapy. However, it remains to be elucidated how ATPase activity of MCM8-9 promotes the functional activity of MRN. Considering that DNA helicases utilize ATP hydrolysis as an energy source^40^, MCM8-9 may function as a helicase in its support of MRN. Alternatively, the physical association of MCM9 with MRE11 is impaired by the mutations in the WA or B motifs of MCM9 (Fig. 4f), suggesting that ATP binding or hydrolysis is important for the protein–protein interaction between these two complexes.

Although MCM8-9 has been shown to associate weakly with RAD51 (ref. 17), the results we present here argue against that being the primary function of MCM8-9 in the loading of RAD51. Instead, MCM8-9 is clearly involved in preparing the ssDNA substrate that eventually loads RAD51.

Impaired HR makes cancers more sensitive to ICL-inducing agents. We show that a naturally occurring homozygous deletion of the *MCM9* sensitizes a cancer cell line to ICL reagents and leads to the HR defect (Fig. 6). The expression level of the MCM9 protein is correlated to the cisplatin resistance of some prostate cancer cell lines (Fig. 6f,g) and a cancer-derived mutation of MCM8 inactivated MCM8 (Fig. 7a). Thus, the deletion, point mutation or reduced expression of *MCM8* or *MCM9* in cancers that we report here should be tested in patients as a biomarker for predicting a cancer's sensitivity to ICL-inducing agents such as cisplatin or mitomycin C. As a promoter of HR, *MCM8* or *MCM9* may serve as a bona fide tumour suppressor similar to other genes important for HR, *BRCA1* and *BRCA2*. A cancer-derived point mutation and a naturally occurring SNP on *MCM8* associated with POF diminish the functional activity of MCM8 (Fig. 7). Together, with a very recent study that appeared while this paper was under review^31^, our results explain how the SNP in *MCM8* impairs HR, an essential function in the germ line, and thus could lead to a genetically determined POF syndrome.

## Methods

### Cell lines and siRNAs

U2OS, ER-*Asi*SI U2OS, HeLa DR13-9 and HEK293T cells were grown in Dulbecco's modified Eagle's medium (Thermo Scientific) with 10% fetal bovine serum (FBS; Sigma-Aldrich) and penicillin/streptomycin (1%, Gibco). LNCaP, PC-3 and NCI-H1299 cells were grown in RPMI 1640 (Gibco) supplemented with 10% FBS and 1% penicillin/streptomycin, and DU145 cells were maintained in Minimum Essential Media (MEM) (Gibco) supplemented with 10% FBS and 1% penicillin/streptomycin. WPE1-NB26 cells were maintained in keratinocyte growth medium (Invitrogen). NCI-H2291 cells were maintained with RPMI 1640 supplemented with 1.5 g l^−1^ sodium bicarbonate, 4.5 g  l^−1^ glucose, 10 mM HEPES, 1 mM sodium pyruvate, 10% FBS and 1% penicillin/streptomycin. *MCM9*-null MEF cells were grown in Dulbecco's modified Eagle's medium with 1 mM sodium pyruvate, 10% FBS and 1% penicillin/streptomycin. ER-*Asi*SI U2OS and HeLa DR13-9 were gifts from Tanya T. Paull and J. Parvin, respectively, and the rest were purchased from American Type Cell Culture (Manassas, VA).

The sequences of the siRNAs used in this paper are as follows: siMCM8, 5′-AGAAGACGCUGAGGAUAUA-3′; siMCM9, 5′-GAUGACUUAGUGGAUAGUU-3′; siMRE11, 5′-ACAGGAGAAGAGAUCAACU-3′; siDNA2, 5′-AGACAAGGUUCCAGCGCCA-3′; siEXO1, 5′-CAAGCCUAUUCUCGUAUUU-3′. Lipofectamine RNAi MAX (Invitrogen) was used for transfection of siRNA following the manufacturer's instruction. Cells were incubated overnight with siRNAs + Lipofectamine and harvested after 48 h in fresh medium.

### Establishment of stable cell lines

The siRNA-resistant WT or mutant of MCM8 and MCM9 was inserted into retrovirus vector pBabe-puro and pLHCX (Clontech), respectively. Retroviral vectors, together with retroviral packaging vector, were transfected into 293T cells using Lipofectamin 2000 (Invitrogen) according to the manufacturer's instruction. At 48 h after transfection, viral culture supernatants were harvested and filtered through a 0.45-μm filter, and added to the U2OS or HeLa DR13-9 cells in the presence of 8 μg ml^−1^ polybrene (Sigma-Aldrich). After 48 h of infection, drug selection was carried out with either 100 μg ml^−1^ hygromycin B (Sigma-Aldrich) or 2 μg ml^−1^ puromycin (Sigma-Aldrich) to select retrovirus-infected cells over a 10-day period.

### Immunoblotting and antibodies

For immunoprecipitation, cells were washed with PBS once and lysed by lysis buffer (20 mM Tris-HCl (pH 7.5), 100 mM NaCl, 1% Nonidet P-40 (NP-40), 5% glycerol, 1 mM EDTA, 1 mM MgCl_2_, 1 mM ATP, 1 mM dithiothreitol (DTT), 10 mM NaF, 1 mM Na_3_VO_4_ and protease inhibitors). After sonication and centrifugation at 21,130*g* for 30 min, 3 mg of lysates was incubated with indicated antibodies and pulled down with protein G-conjugated agarose beads (GE Healthcare) in the presence of EtBr (10 μg ml^−1^). For immunoblotting, 50–70 μg of protein was subjected to SDS–polyacrylamide gel electrophoresis analysis. Antibodies for this study were as follows: MCM8 and MCM9 antibodies were raised in rabbits against the N- and C-terminal 100 amino acids of MCM8 and MCM9 proteins, respectively. Anti-MCM8 (Immunoblot (IB), 1:100) and anti-MCM9 (IB, 1:500; Immunoprecipitation (IP), 12 μl per reaction)^17^; anti-RPA70 (IB, 1:1,000; Immunofluorescence (IF), 1:100; NA13; Calbiochem); anti-FLAG (IF, 1:500; F1804; Sigma); anti-α-tubulin (IB, 1:1,000; sc-5286); anti-HA (IB, 1:1,000; IF, 1:200; sc-805) and anti-CHK1 (IB, 1:500; sc-8408) (Santa Cruz Biotechnology); anti-RAD50 (IB, 1:1,000; IF, 1:200; IP, 10 μg per reaction; GTX70228; Genetex); anti-EXO1 (IB, 1:1,000; ab95012) and anti-DNA2 (IB, 1:1,000; ab96488; Abcam); anti-p-CHK1 (Ser317) (IB, 1:1,000; 2344S) and anti-γ-H2AX (S139) (IB, 1:1,000; 2577L; Cell Signaling Technology); anti-MRE11 (IB, 1:8,000; IF, 1:200; NB100-142); anti-NBS1 (IB, 1:8,000; IF, 1:200; NB100-143); and anti-CtIP (IB, 1:1,000; IF, 1:500; NB100-79810; Novus biological). α-Tubulin was used as loading control in most immunoblots. Uncropped images of immunoblots presented in the main paper are provided in Supplementary Fig. 13.

### Immunostaining

Cells were fixed with 4% paraformaldehyde with 0.1% Triton X-100 in PBS for 10 min and permeabilized with 0.5% Triton X-100 in PBS for 3 min at room temperature. Coverslips were incubated in 10% FBS in PBST (0.1% Triton X-100 in PBS) overnight at 4 °C, and then incubated with primary antibody for 2 h. Cells were washed three times with PBST and incubated with either Alexa Fluor 555 anti-rabbit (1:1,000, A21429; Life Technologies) or Alexa Fluor 488 anti-mouse (1:500, A11029; Life Technologies) immunoglobulin G secondary antibody. Cells were mounted with a solution containing 4′,6′-diamidino-2-phenylindole (Vector Laboratories, Inc.) before being examined under a microscope. To detect ssDNA, U2OS cells were pre-labelled with 10 μM BrdU for 24 h before the first transfection of siRNA, and BrdU was maintained until 48 h harvest after transfection. MEFs cells were incubated with 10 μM BrdU for 48 h. In most experiments, 40 μM cisplatin was added for 4 h before harvest and pre-extraction was performed using 0.5% Triton X-100 in PBS for 1 min only for BrdU staining (1:100, 555627; BD Pharmingen). A Zeiss Axio observer A-1 equipped with an EC Plan-Apochromat ( × 63/1.4 oil) and Axio-vision software were used to obtain and analyse images, respectively. Cells having >20 foci per cell were counted. Brightness and contrast of obtained images were adjusted using Photoshop 7.0 (Adobe).

### ChIP assay

HeLa DR13-9 cells stably expressing MCM9 (WT or mutant in WA/WB motifs) were fixed with 1% formaldehyde for 10 min at room temperature and incubated with 0.125 M glycine for 5 min. Cells were lysed by lysis buffer (50 mM Tris-HCl (pH 8.0), 10 mM EDTA (pH 8.0), 0.1% SDS and protease inhibitor cocktail (Roche)) on ice for 10 min followed by sonication (30 s-on/30 s-off, eight times at 11% amplitude using Sonic Dismembrator model 500 (Fisher Scientific)). Lysates were diluted to one-tenth using dilution buffer (50 mM Tris-HCl (pH 8.0), 150 mM NaCl, 1% Triton X-100, 0.1% SDS and protease inhibitor cocktail) and incubated with protein G-conjugate dynabeads (Invitrogen), which were bound with the indicated antibody (anti-RPA70 (5 μl), anti-RAD50 (3 μl), anti-MRE11 (2 μl), anti-NBS1 (2 μl) or anti-CtIP (2 μl) was mixed with 20 μl dynabeads for each reaction). After sequential washing was done by RIPA-150 (50 mM Tris-HCl (pH 8.0), 150 mM NaCl, 1 mM EDTA, 1% Triton X-100, 0.1% SDS and 0.1% sodium deoxycholate), RIPA-500 (same components with RIPA-150 except for 500 mM NaCl), LiCl buffer (10 mM Tris-HCl (pH 8.0), 0.15 M LiCl, 1 mM EDTA, 0.5% NP-40 and 0.5% sodium deoxycholate) and Tris-EDTA (TE) buffer, the pellets were incubated in elution buffer (10 mM Tris-HCl (pH 8.0), 300 mM NaCl and 5 mM EDTA) and 0.5% SDS at 65 °C for 8 h. The eluted DNA was recovered by phenol:chloroform extraction and analysed by qPCR using ABI 7300 real-time PCR system (Applied Biosystems). Primers sequences used for qPCR were as follows: I-SceI cut site (F1, 5′-TACGGCAAGCTGACCCTGAA-3′ ; R1, 5′-GAAGTCGTGCTGCTTCATGT-3′) and 2 kb upstream of cut site (control) (F2, 5′-GCCCATATATGGAGTTCCGC-3′; R2, 5′-CCCTATTGGCGTTACTATGG-3′)

### Quantitative resection assay using the ER-*Asi*SI system

A quantitative resection assay^22^ to quantitatively measure ssDNA intermediates was performed. ER-*Asi*SI U2OS cells were mixed with 37 °C 0.6% low-melting temperature agarose (FMC Bioproducts) in PBS at a concentration of 6 × 10^6^ cells per ml. Fifty microlitres of cell suspension was solidified as an agar ball by dropping on a Parafilm (Bemis). The agar ball was serially treated by 1 ml of EDTA-sarcosine-proteinase (ESP) buffer (0.5 M EDTA, 2% *N*-lauroylsarcosine, 1 mg ml^−1^ proteinase K and 1 mM CaCl_2_ (pH 8.0)) and high salt (HS) buffer (1.85 M NaCl, 0.15 M KCl, 5 mM MgCl_2_, 2 mM EDTA, 4 mM Tris and 0.5% Triton X-100 (pH 7.5)) for 20 h per each time at 16 °C with rotation, followed by washing with 1 ml of phosphate buffer (8 mM Na_2_HPO_4_, 1.5 mM KH_2_PO_4_, 133 mM KCl and 0.8 mM MgCl_2_ (pH 7.4)) for 6 × 1 h at 4 °C with rotation. After heating of the agar ball at 70 °C for 10 min, it was diluted 15-fold with 70 °C ddH_2_O and mixed with 2 × NEB restriction enzyme buffer 4. Sixty microlitres of genomic DNA sample was digested or mock-digested with 60 units of restriction enzyme, BsrGI (New England Biolabs) at 37 °C overnight. Three microlitres of mock- or BsrGI-digested samples was used as templates in 20 μl of qPCR reaction containing 0.5 μM of each primer and ABsolute Blue qPCR SYBR Green ROX (Thermo Scientific) using ABI 7300 real-time PCR system (Applied Biosystems).

### HR assay

HeLa DR13-9 cells having stable expression of siRNA-resistant MCM9 were used for HR assay^17^,^21^. siMCM9 was transfected for 24 h, followed by transfection with the I-SceI expression vector pCβASce^41^ using Lipofectamine2000 (Invitrogen) for 48 h. For HR assay of NCI-H1299 and NCI-H2291 cells, each 2 μg of the DSB recombination reporter pDR-GFP-hprt^42^ and pCβASce was mixed and co-transfected into those cell lines using Lipofectamine2000 for 48 h. In the mean time, 2 μg of the modified enhanced green fluorescent protein (EGFP) expression vector (NZ-EGFP)^43^ was separately transfected into those cells to evaluate the transfection efficiency. The green fluorescent protein (GFP)-expressing cells were counted in flow cytometric analysis (BD FACSCalibur). The HR efficiency was calculated by normalizing the GFP% to the transfection efficiency in each cell lines.

### Purification of *Xenopus* MCM8-9 from Sf9 cells

Sf9 cells (1 × 10^6^ cells per ml, 2 l), grown in suspension culture in Grace's medium supplemented with 10% FBS at 27 °C, were infected with baculoviruses expressing combinations of His-tagged *Xenopus* MCM8 with WT or WA mutant of His-tagged MCM9. After 2 days at 27 °C, cells were harvested by centrifugation at 1,200*g* for 15 min at 4 °C, and washed once with PBS. The pellets were resuspended in 50 ml of lysis buffer (20 mM Tris-HCl (pH7.4), 100 mM NaCl, 0.1% NP-40, 10 mM MgCl_2_, 5 mM 2-mercaptoethanol, 10 mM NaF, 2 mM Na_2_VO_3_, 10% glycerol and protease inhibitor). The cells were kept on ice for 10 min and then lysed by sonication (10 s-on/10 s-off, two times at 20% amplitude using Sonic Dismembrator model 500 (Fisher Scientific)). The lysates were collected by centrifugation at 13,000 r.p.m. for 30 min at 4 °C. The cleared lysate was mixed with Ni-NTA agarose beads (Qiagen) for 1 h and the beads were washed with lysis buffer supplemented with 20 mM imidazole. Bound proteins were eluted with lysis buffer supplemented with 200 mM imidazole, and then diluted fivefold with dilution buffer (20 mM Tris-HCl (pH 7.4), 0.1% NP-40, 10 mM MgCl_2_, 5 mM 2-mercaptoethanol and 10% glycerol). The eluted proteins were applied to Hitrap Heparin HP column (Amersham Pharmacia Biotech) in the fast protein liquid chromatography (FPLC), and 1-ml fractions were collected in elution buffer with a linear gradient from 50 mM to 1 M NaCl. The peak fractions were pooled and desalted by a PD-10 desalting column (GE Healthcare Life Sciences). These were next applied to a Hitrap Q HP column (Amersham Pharmacia Biotech) in the FPLC, and 1-ml fractions were collected in elution buffer with a linear gradient from 100 mM to 500 mM NaCl. Final peak fractions were concentrated by using Amicon Ultra Centrifugal filters (Millipore), and elution buffer was changed into storage buffer (20 mM Tris-HCl (pH 7.4), 100 mM NaCl, 0.01% Triton X-100, 10% glycerol and 1 mM DTT) by a PD-10 desalting column. To check the purity in each step of purifications, small aliquots of each fraction was run on a 8% SDS–PAGE gel and silver-stained using a Silver Stain Kit (Pierce).

### Immunodepletion of insect MRE11 from purified MCM8-9

The protein G-conjugated agarose beads conjugated with anti-MRE11 antibody were incubated with purified proteins at 4 °C overnight and separated from supernatant. One-fifth of the input protein before immunodepletion (B) or after immunodepletion (A) and the total immunoprecipitate (I) were loaded on 8% SDS–PAGE gel and detected by anti-human MRE11 antibody in the western blot technique.

### *In vitro* nuclease assay

To purify FLAG-tagged NBS1 or MCM9 protein, we used U2OS and HeLa DR13-9 cell lines that stably express FLAG-NBS1 and HA-MCM9, respectively. Cells were transfected with siGL2, siMCM8 or siMCM9 for 48 h and each of the lysates were obtained by the same procedures as used for immunoprecipitation assays. Ten microlitres of anti-FLAG M2 affinity gel (A2220, Sigma) or EZview Red Anti-HA Affinity beads (E6779, Sigma) was incubated with 1.5 or 3 mg of lysate overnight at 4 °C, and washed with lysis buffer four times and then with elution buffer (62.5 mM HEPES (pH 7.4), 62.5 mM KCl, 5% glycerol, 1 mM DTT and 50 μg ml^−1^ BSA) accompanied by rotation for 5 min each wash. Finally, proteins were eluted by incubation with 20 μl of elution buffer supplemented with 150 μg ml^−1^ FLAG or HA peptide (sigma). The *in vitro* nuclease assay was done as previously published with slight modifications^8^,^36^. pUC19 (1.88 or 3.75 nM) plasmid linearized by HindIII was mixed with the indicated amount of purified MCM8-9 or purified MRN (FLAG-NBS1) in reaction buffer (20 mM HEPES (pH 7.5), 0.1 mM DTT, 5 mM MnCl_2_, 2 mM ATP, 0.1 mg ml^−1^ BSA and 0.05% Triton X-100) for 15 or 30 min at 37 °C. Alternatively, 100 ng of circular ØX174 ssDNA virion DNA (New England Biolabs) was mixed with purified MRN (FLAG-NBS1) in reaction buffer (30 mM Tris-HCl (pH 7.5), 1 mM DTT, 25 mM KCl, 200 ng acetylated BSA, 0.4% DMSO and 5 mM MnCl_2_) in the presence of 8 mM ATP for indicated times (Fig. 5a) at 37 °C. The reaction was stopped by adding 0.2 mg ml^−1^ proteinase K, 50 mM EDTA and 3% SDS (final concentrations) by incubating for 20 min, and then the reaction products were loaded on 1% native agarose gel. DNA was stained with EtBr, visualized by FluorChem Q (Proteinsimple) and quantified by ImageJ software.

### ATPase assay

The indicated amount of purified MCM8-9 was incubated with reaction buffer containing 25 mM HEPES-NaOH (pH 7.5), 50 mM sodium acetate, 5 mM magnesium acetate, 1 mM DTT, 0.1 mg ml^−1^ BSA and 1.5 nmol of [γ-^32^P]ATP (6,000 Ci mmol^−1^, Perkin-Elmer) at 60 °C for 1 h. Two microlitres of each sample (15 μl) was spotted onto a thin-layer chromatography (TLC) plate (Sigma-Aldrich) followed by development in 1 M formic acid and 0.5 M LiCl, and ATP hydrolysis was visualized by autoradiography using X-ray film (Kodak).

### Cell survival assay

U2OS cell lines, which stably expressed WT or mutants of the WA or WB motif of MCM9, and various prostate cancer cells were used for the MTT (3-(4,5-dimethylthiazol-2-yl)-2,5-diphenyltetrazolium bromide) assay. The indicated amount of cisplatin (Sigma-Aldrich) was incubated with cells for 24 h, and cell viability was measured by MTT assay, following the manufacturer's protocol (Promega, CellTiter 96 Non-Radioactive Cell Proliferation Assay (MTT)). To deplete endogenous MCM9 from stable U2OS cells, siMCM9 was transfected into cells at 24 h before cisplatin treatment. For the clonogenic assay, U2OS cell lines having WT or mutants of MCM8 or MCM9 were transfected with the indicated siRNA and plated on a six-well plate after 24 h. The indicated concentration of cisplatin was added for 5 h, beginning at 42 h after siRNA transfection. At day 5 after DNA damage, the colonies were stained with crystal violet. NCI-H2291 cells were plated and transfected with the indicated plasmid using Lipofectamine2000 (Invitrogen). After transfection, 10,000 cells of each transfectant were plated into one well of a six-well plate, and incubation with 2 μM of cisplatin was started at 36 h after transfection. Cisplatin was washed out with fresh medium after 24 h. The colonies were stained with crystal violet at 12 days after DNA damage and quantified using Gene Tools software (Syngene).

## Additional information

**How to cite this article:** Lee, K. Y. *et al.* MCM8-9 complex promotes resection of double-strand break ends by MRE11-RAD50-NBS1 complex. *Nat. Commun.* 6:7744 doi: 10.1038/ncomms8744 (2015).

## Supplementary Material

Supplementary Information Supplementary Figures 1-13

## Figures and Tables

**Table 1:** A list of some of the cancers reported to have homozygous deletion of *MCM9* locus from copy-number variation (CNV) experiments collated in CBioPortal.

**Cancers with homozygous deletion of** ***MCM9*** **locus**	
Cancers	Case ID
Breast invasive carcinoma	TCGA-AR-A0TW
	TCGA-AR-A1AU
Glioblastoma	TCGA-06-5415
	TCGA-26-1442
	TCGA-02-0058
Kidney renal clear cell carcinoma	TCGA-CZ-5460
Ovarian serous cystadenocarcinoma	TCGA-24-2267
	TCGA-36-2543
Prostate adenocarcinoma	PCA0119
	PCA01555
	PCA0126
Bladder urothelial carcinoma	TCGA-H4-A2HQ
	TCGA-HQ-A20E
Head and neck squamous cell carcinoma	TCGA-CQ-5329
Prostate adenocarcinoma	TCGA-CH-5748
	TCGA-EJ-5494
	TCGA-EJ-5505
	TCGA-EJ-5509
	TCGA-G9-6369
Skin cutaneous melanoma	TCGA-D9-A149
	TCGA-EE-A2MK
