# Supplementary material for: MCM8-9 complex promotes resection of double-strand break ends by MRE11-RAD50-NBS1 complex
Source: Nat Commun. 2015 Jul 28;6:7744. doi: 10.1038/ncomms8744 (PMC4525285; doi:10.1038/ncomms8744)
Supplement: Supplementary Information — Supplementary Figures 1-13 [file ncomms8744-s1.pdf]

Supplementary Figure 1

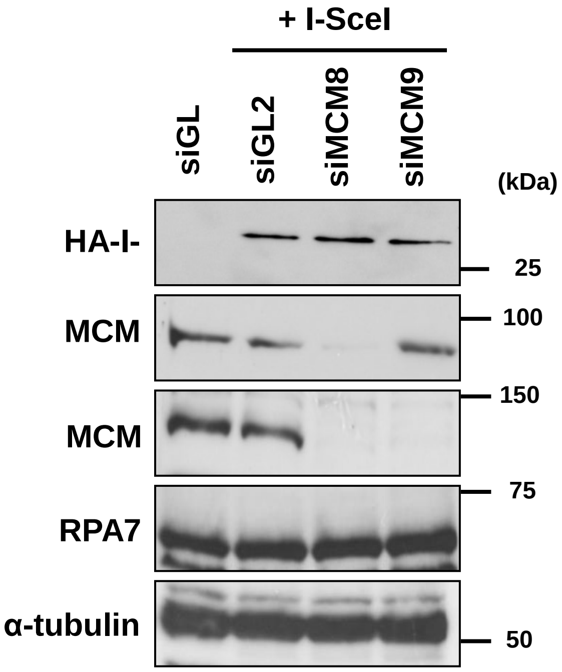

Supplementary Figure 1 | Depletion of MCM8 or MCM9 does not decrease transient expression of I-SceI . Immunoblots of lysates from HeLa DR13-9 cells transfected with indicated siRNAs and I-SceI expression plasmid, pCβASce. I-SceI was detected by anti-HA antibody.

Supplementary Figure 2

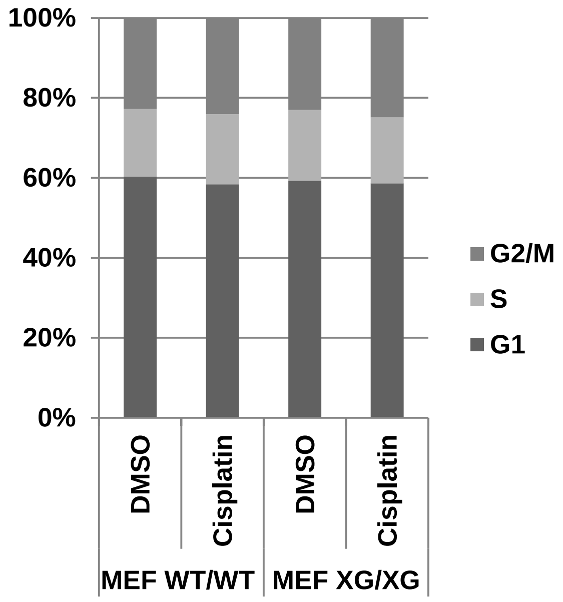

Supplementary Figure 2 | MCM9 mutation does not affect on cell cycle progression under cisplatin treatment. MEF cells with wild type *MCM9* (WT/WT) or homozygously mutated for *MCM9* (XG/XG) were treated by 40 μM cisplatin for 4 hr and then, cell cycle profile was analyzed by propidium iodide staining using FACSCalibur Flow Cytometry System.

Supplementary Figure 3

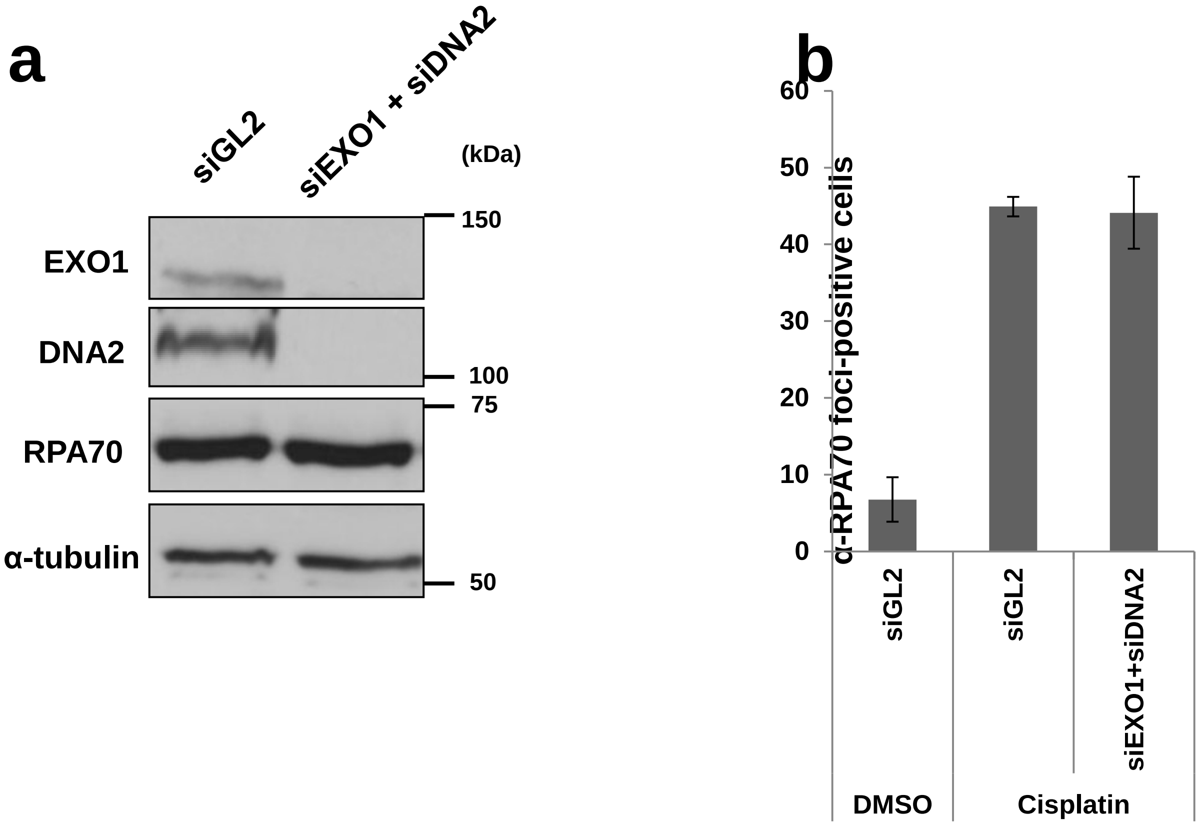

Supplementary Figure 3 | Double-knockdown of EXO1 and DNA2 does not suppress cisplatin-induced RPA foci-positive cells. (**a**) siEXO1 and siDNA2 were co-transfected into U2OS cells for 48 hr and the expression levels of the proteins were detected by immunoblotting. DMSO or 40 μM cisplatin was added for 4 hr before harvest. (**b**) Quantification of α-RPA70 foci-positive cells. Error bars represent SD of the mean from triplicates.

Supplementary Figure 4

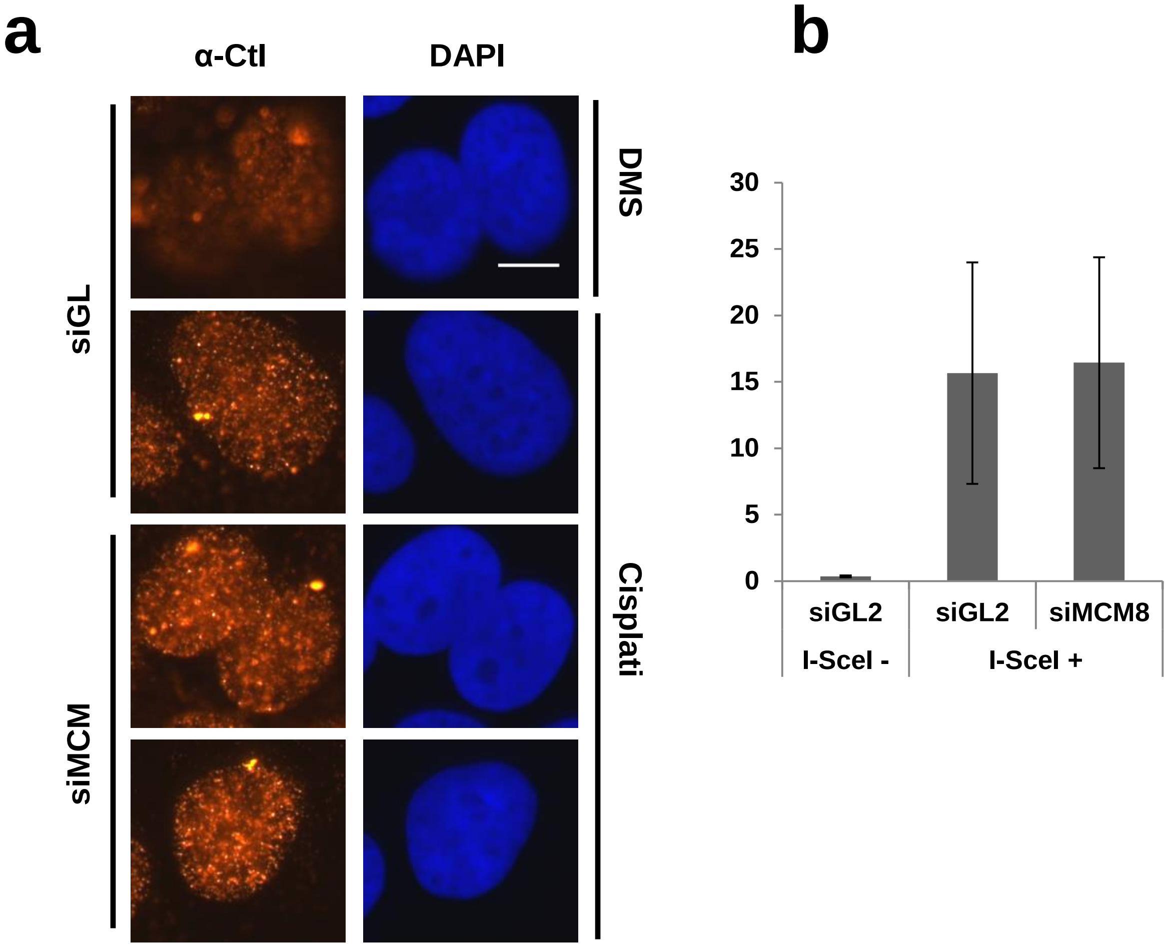

Supplementary Figure 4 | MCM8-9 is not responsible for the localization of CtIP. (**a**) siGL2 or siMCM8 was transfected in U2OS cells for 48 hr and cells were stained with anti-CtIP antibody. DMSO or 40 µM cisplatin was added for 4 hr before harvest. Scale bar, 10 µm. (**b**) CtIP attachment at I-SceI cut site in MCM8-depleted cells seen by ChIP with anti-CtIP antibody using HeLa DR13-9 cells. Fold signal at I-SceI cut site relative to control site 2 kb upstream of cut site. Error bars represent SD of the mean from triplicates.

Supplementary Figure 5

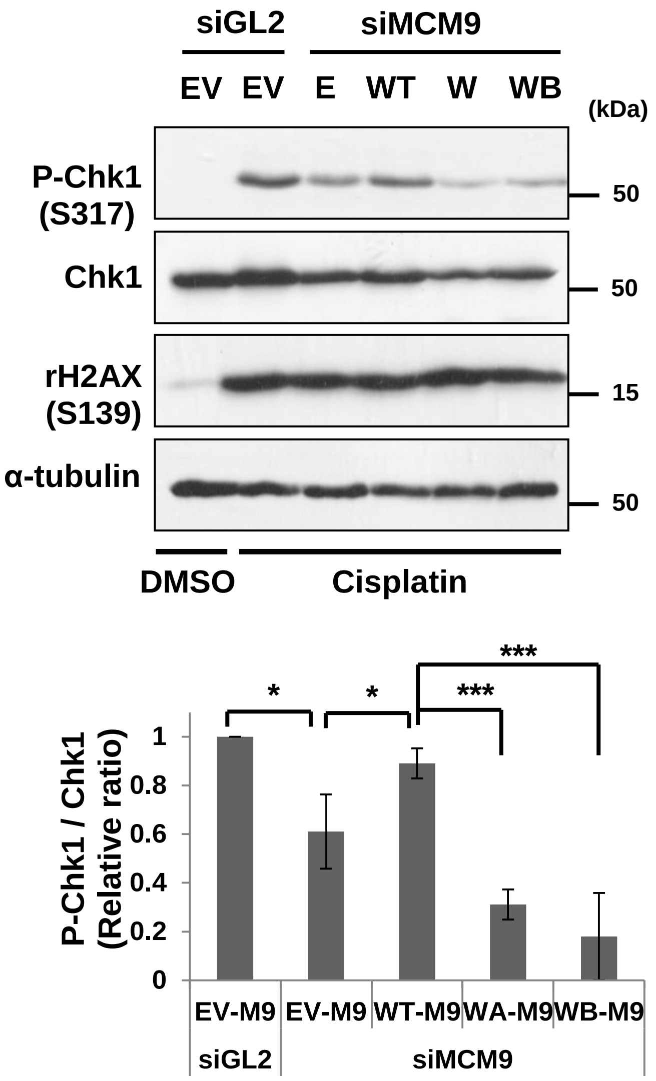

Supplemental Figure 5 | WA- or WB-mutant of MCM9 decreases checkpoint activation after knockdown of endogenous MCM9. U2OS cells supported by wild type, Walker A-(WA) or Walker B (WB)-mutants of MCM8 or 9 were treated with 40 μM cisplatin for 1 hr after knockdown of endogenous MCM9. Immunoblots using indicated antibodies are shown (top) and the relative ratio between phosphorylated Chk1 and total Chk1 was obtained by normalizing the ratio from three independent experiments to that of siGL2-treated cells (bottom). Band intensity was quantified using Imagej software. Phosphorylated residue on the protein shown in brackets. Error bars represent SD of the mean from triplicates. ***, $P < 0.005$; *, $P < 0.05$. Student's *t*-test.

Supplementary Figure 6

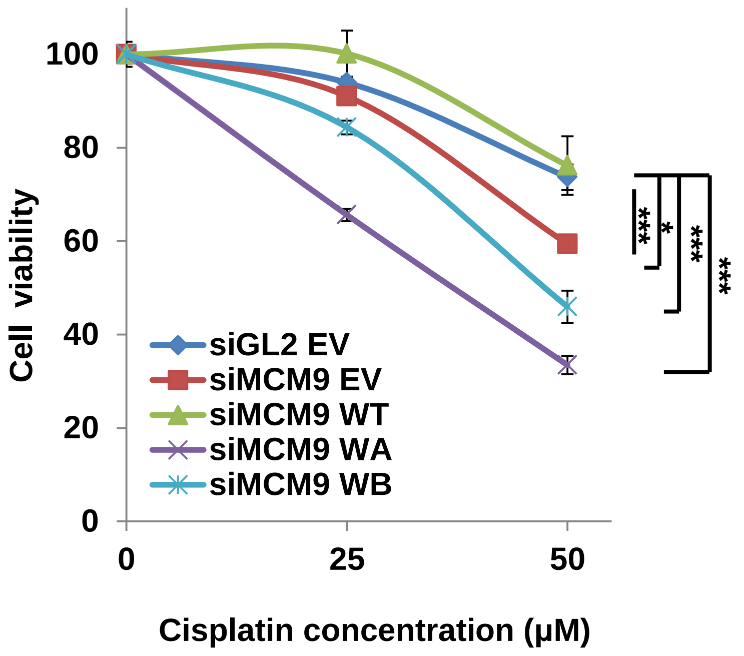

Supplementary Figure 6 | ATPase activity of MCM9 is required for cellular resistance to cisplatin. siMCM9 was transfected into the indicated cell lines for 48 hr to deplete endogenous MCM9 and cell viability was measured by MTT assay by treating indicated concentration of cisplatin for 24 hr before MTT reaction. Mean ± SD from three independent experiments. Error bars represent SD of the mean from triplicates. ***, $P < 0.005$; *, $P < 0.05$. Student's *t*-test.

Supplementary Figure 7

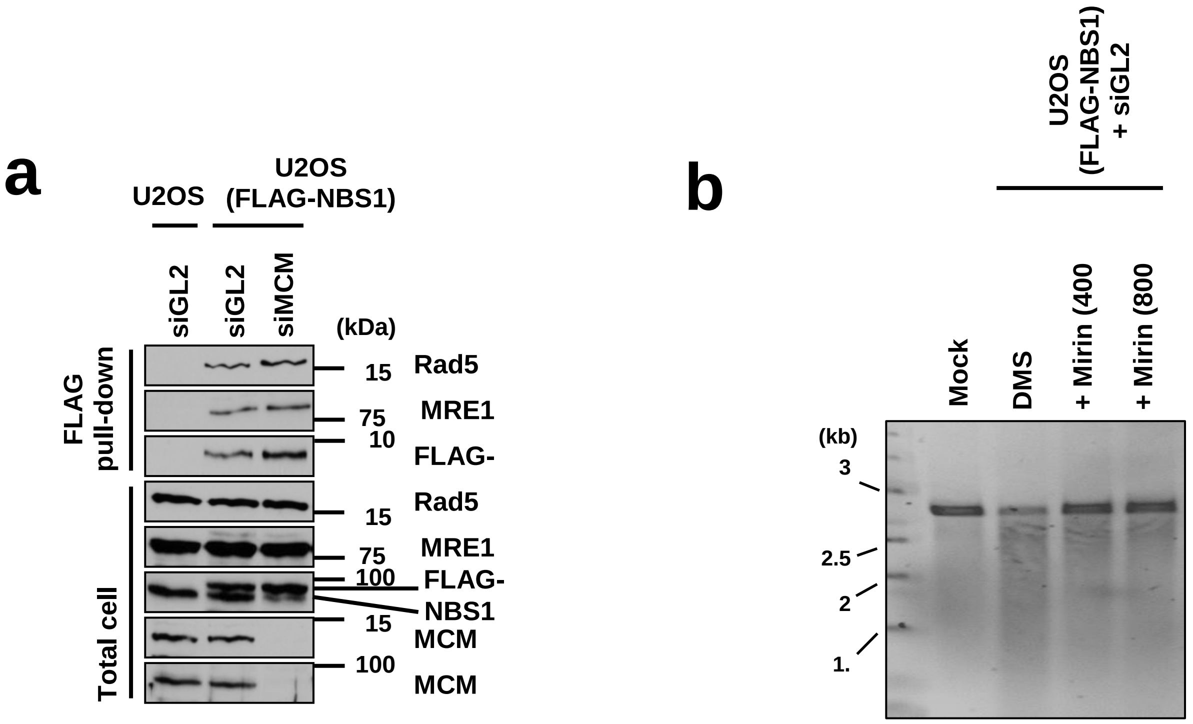

Supplementary Figure 7 | Related to Fig. 5A. (**a**) The amount of purified MRN complex is not decreased by siMCM8. Immunoblots of cell lysates and FLAG immunoprecipitate from U2OS cells stably expressing FLAG-NBS1 for *in vitro* nuclease assay. (**b**) MRE11 inhibitor, mirin, inhibits the nuclease activity of purified MRN from U2OS cells stably expressing FLAG-NBS1. The indicated concentration of mirin was incubated with 1.88 nM linearized pUC19 and same amount of purified MRN used in the experiment of fig. 5A for 30 min at 37 ºC for *In vitro* nuclease assay.

Supplementary Figure 8

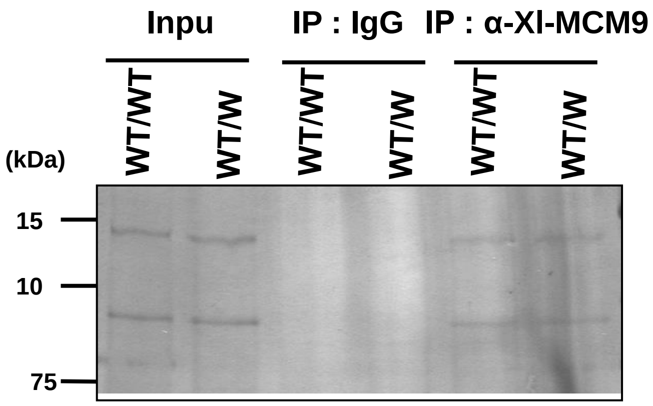

Supplementary Figure 8 | Xenopus MCM8-MCM9 form complex even after mutation of Walker A motif of MCM9. IP with anti-xenopus MCM9 antibody using 100 ng of purified recombinant WT/WT or WT/WA MCM8-9 complexes. The immunoprecipitates were loaded on 8% SDS-PAGE gel and stained with silver. 50 ng protein of each sample was loaded as Input.

Supplementary Figure 9

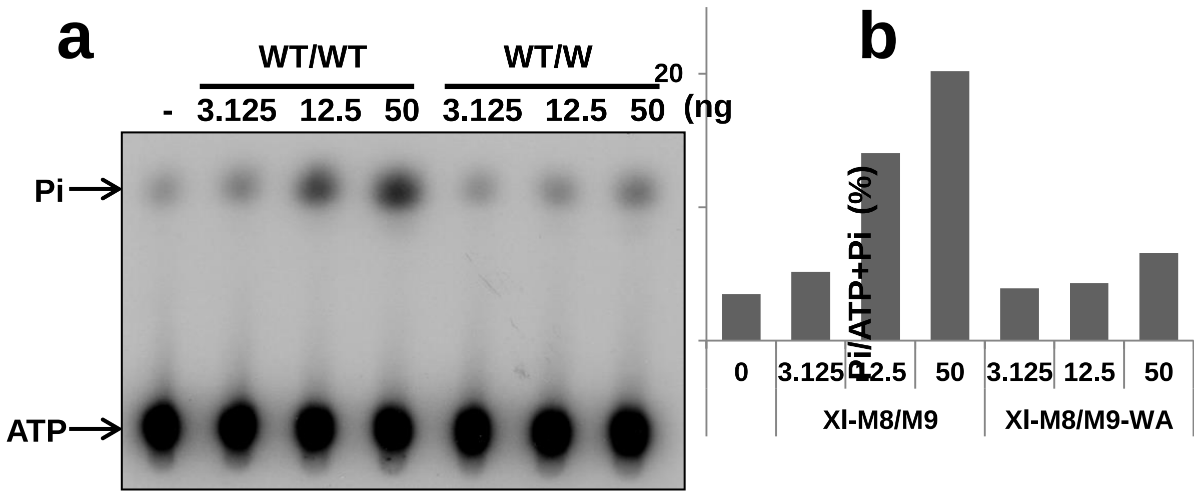

Supplementary Figure 9 | ATPase activity of recombinant MCM8-9. (**a**) ATPase assay was performed using indicated amounts of the recombinant MCM8-9 as described in **Methods** section. (**b**) ATP hydrolysis by recombinant protein was calculated by dividing the count of hydrolyzed phosphate (Pi) to the total count of non-hydrolyzed ATP plus hydrolyzed phosphate (ATP + Pi) measured by liquid scintillation counter.

Supplementary Figure 10

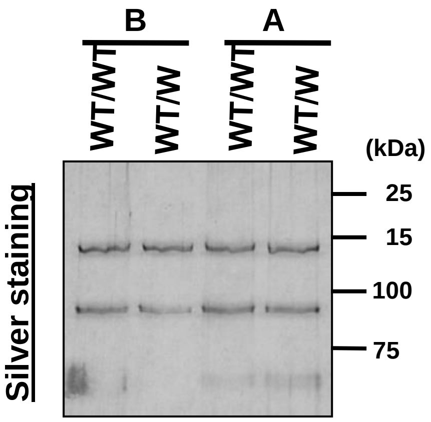

Supplementary Figure 10 | The amount of recombinant MCM8-9 is not decreased by MRE11 immunodepletion. Recombinant purified proteins of WT/WT or WT/WA before (B) and after (A) MRE11 immunodepletion was applied to 8% SDS-PAGE gel and silver-stained.

Supplementary Figure 11

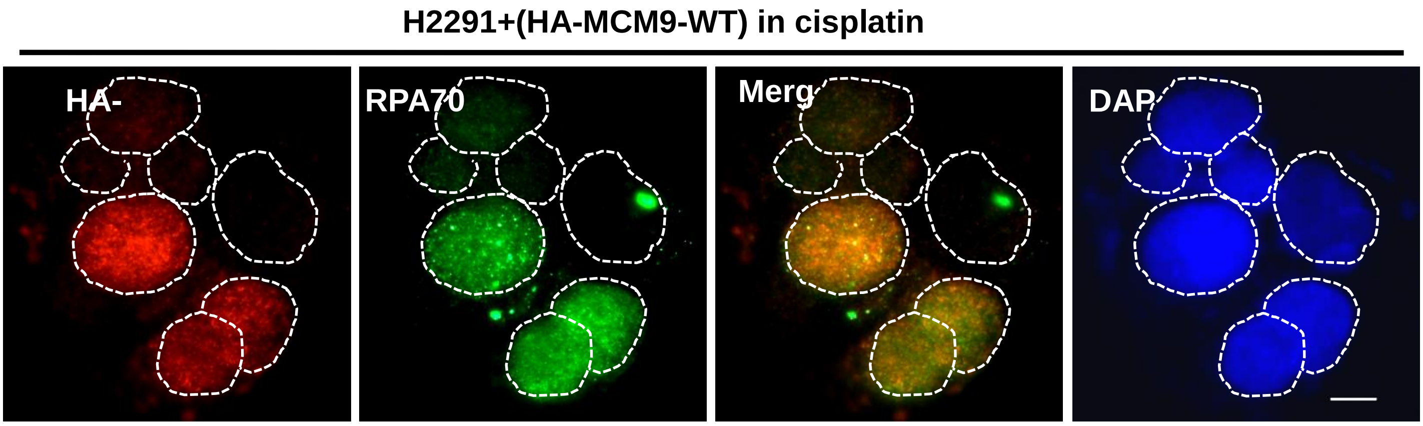

Supplementary Figure 11 | Restoration of cisplatin-induced RPA70 focus formation in NCI-H2291 by transient transfection of HA-MCM9. Overexpressed MCM9 was detected by anti-HA antibody. Scale bar, 10 μm.

Supplementary Figure 12

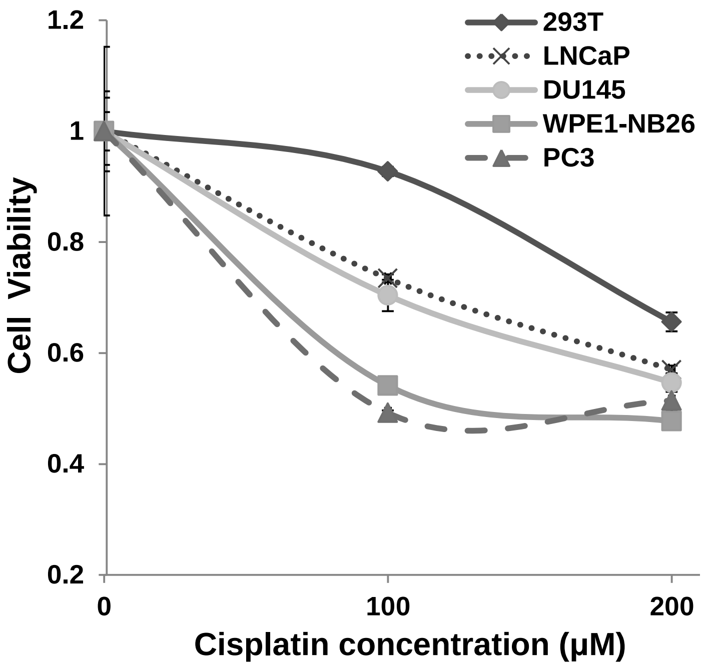

Supplementary Figure 12 | Cell viability after 24 hr of indicated concentrations of cisplatin was measured by MTT assay. Error bars represent SD of the mean from triplicates. IC75 is the concentration of cisplatin at which viability is reduced to 75%.

Supplementary Figure 13

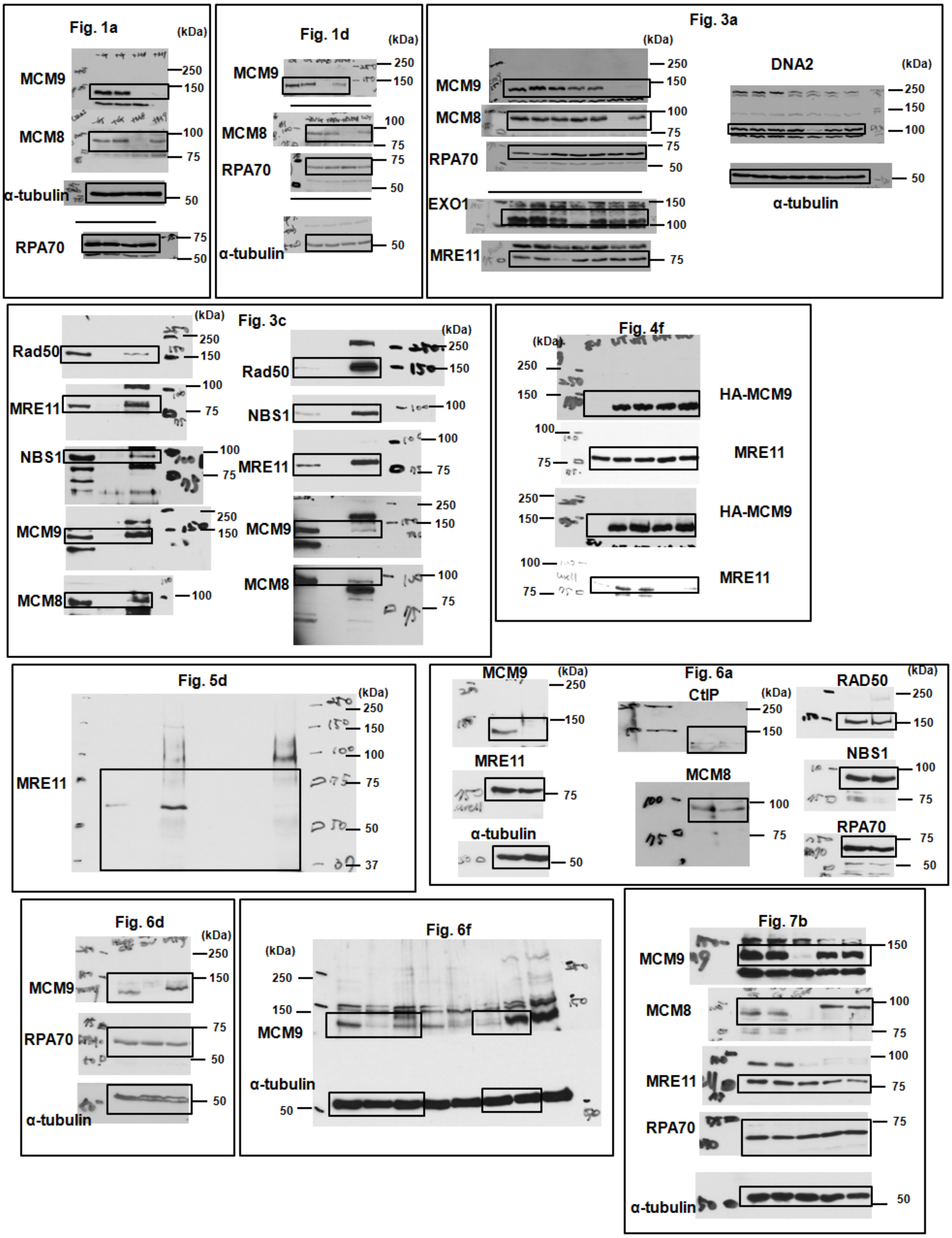

Supplementary Figure 13 | Uncropped original images of immunoblotting results for the main figures.
